# Extremely elevated IL-18 levels may help distinguish systemic-onset juvenile idiopathic arthritis from other febrile diseases

**DOI:** 10.1590/1414-431X20165958

**Published:** 2017-02-16

**Authors:** Y. Xia, P. Cui, Q. Li, F. Liang, C. Li, J. Yang

**Affiliations:** 1 Department of Rheumatology, Shenzhen Children’s Hospital, Shenzhen, Guangzhou, China; 2 Department of Rheumatology, Children’s Hospital of Chongqing Medical University, Chongqing, China

**Keywords:** Systemic-onset juvenile idiopathic arthritis (sJIA), Serological markers, IL-18, S100A8/S100A9

## Abstract

The aim of this research was to explore whether IL-18 can be a serological marker for the diagnosis of systemic-onset juvenile idiopathic arthritis (sJIA). A total of 23 sJIA patients (13 males, median age 8.2), 20 acute lymphoblastic leukemia (ALL) patients, 18 patients with severe infections (SIF), 26 Kawasaki disease (KD) patients, 18 juvenile idiopathic arthritis (JIA) patients, and 25 healthy control patients were selected for this study. Enzyme-linked immunosorbent assays (ELISAs) were used to determine the serum concentrations of the S100A8, S100A9, and IL-6 proteins. The serum IL-18 levels were detected by a cytometric bead array (CBA). The serum IL-6 concentrations in various disease groups were significantly higher than that in the healthy control group. The IL-6 concentrations exhibited no significant difference between disease groups. The S100A8 level in the sJIA group was significantly higher than those of the ALL, JIA, and healthy control groups but showed no significant difference compared to the SIF and KD groups. The S100A9 serum concentration in the sJIA group was significantly higher than those in the ALL and healthy control groups and exhibited no significant difference from the SIF, KD, and JIA groups. The IL-18 level of the sJIA group was significantly higher than that of the other febrile disease groups. The IL-18 serum concentration may be used as a biological serum marker to distinguish sJIA from other febrile diseases.

## Introduction

Systemic-onset juvenile idiopathic arthritis (sJIA) is a type of auto-inflammatory disease that shows clinical manifestations such as fever, rash, hepatosplenomegaly, serositis, and synovitis (1). These clinical manifestations vary greatly among patients, and some children do not exhibit arthritis symptoms at the early stage and therefore do not meet the diagnostic criteria of JIA (2). In addition, for a diagnosis of atypical sJIA, the possibility of febrile diseases such as infection, malignant tumor, and atypical Kawasaki disease (KD) must be excluded. Identifying highly sensitive and specific serological biomarkers is a prominent research topic in the early diagnosis of sJIA. It has been reported that the serum concentrations of S100A8, S100A9, and IL-18 are increased in sJIA patients (3–5), but no study has been conducted to determine whether these biomarkers can be used in the diagnosis of sJIA. In the present study, the serum concentrations of IL-6, IL-18, S100A8, and S100A9 were measured and comparatively analyzed in pediatric patients with sJIA and other diseases that can be easily misdiagnosed as sJIA, including acute lymphoblastic leukemia (ALL), severe infections (SIF), KD, and juvenile idiopathic arthritis (JIA) with multiple joint involvement. The feasibility of using different inflammatory factors as serological biomarkers for the diagnosis of sJIA was investigated.

## Material and Methods

A total of 23 sJIA patients who visited the Children's Hospital of Chongqing Medical University and the Pediatric Research Institute of Shenzhen Children^’^s Hospital between July 2013 and September 2014 were selected for this study, including 13 males and 10 females, with a median age of 8.21 years old (4.56-10.08 years old).

The diagnoses were performed in accordance with the ILAR 2001 diagnostic classification criteria (2). Other disease diagnoses were excluded, and all patients were followed-up for more than 6 months. None of the pediatric patients had taken glucocorticoids or other immunosuppressive agents prior to the collection of specimens. Blood samples of patients in the ALL and KD groups were collected during the acute stage, and the patients had not begun the use of hormones, chemotherapy drugs, and gamma globulin protein therapy. The diagnosis of KD was performed in accordance with the standards developed by the Japanese Kawasaki Disease Research Committee. The diagnosis of ALL was confirmed by a bone marrow biopsy. Patients in the SIF group consisted of children hospitalized in the pediatric intensive care unit, including 14 patients with purulent meningitis (7 cases with blood culture showing Gram negative bacilli, 2 cases of *Ochrobactrum anthropi*, 2 cases of *Escherichia coli*, and 3 cases of *Streptococcus pneumoniae*); 7 patients with pulmonary infection (3 cases with alveolar lavage fluid culture revealing positive hemolytic *Staphylococcus*, 2 cases of *Staphylococcus aureus*, 1 case of *Pseudomonas aeruginosa*, and 1 case of *Streptococcus pneumoniae*); 1 patient with diffuse peritonitis with ascites culture showing *Klebsiella pneumoniae*; and 1 patient with acute hemorrhagic necrotizing enteritis with peritoneal drainage fluid culture indicating *E. coli*. All samples were tested immediately after collection. A total of 25 healthy subjects underwent physical examinations and were enrolled in the healthy control group.

This study was approved by the Ethics Committee of the Affiliated Children's Hospital of Chongqing Medical University and the Shenzhen Children's Hospital. Informed consent was obtained from all parents of the patients included in the study.

### Methods

Venous blood (2 mL) was collected, and the anticoagulant disodium ethylenediaminetetraacetic acid (EDTA-Na_2_) was added under sterile conditions. The sample was centrifuged at 600 *g* for 5 min at 4^o^C, and the supernatant plasma was collected and preserved in a -80°C freezer. After the pre-separated plasma samples were retrieved and thawed at room temperature, the IL-6, S100A9, and S100A8 protein levels were detected by an enzyme-linked immunosorbent assay (ELISA) following the manufacturer’s instructions (IL-6: eBioscience, USA; S100A8 and S100A9: CycLex, Japan). A cytometric bead array (CBA) was used to detect the serum IL-18 level according to the instructions of the CBA kit (eBioscience). The expression of IL-18 in the serum was measured by flow cytometry (FACSCanto, BD, USA) and analyzed using FlowCytomix Pro software (Bender MedSystemsGermany).

### Statistical analyses

The data were analyzed with SPSS17.0 software (USA), and all datasets were tested for normality. Differences in data between multiple groups were analyzed using one-way ANOVA. Pairwise comparisons between groups were analyzed with Dunnett’s T3 test. M (Q1, Q3) and H (Kruskal-Wallis) tests were used to analyze data with non-normal distributions. The sensitivity and specificity of the diagnostic markers were analyzed by comparing the area under the receiver operating characteristic (ROC) curve, and the cutoff values of the markers for differential diagnosis were further analyzed.

## Results

### Clinical data analyses of pediatric sJIA patients

The erythrocyte sedimentation rate (ESR) of sJIA patients was significantly higher than the JIA group (P<0.05). The cAMP receptor protein (CRP) level of the sJIA patients was higher than the ALL group (P<0.05). The platelet count in the sJIA group was also significantly higher than the ALL group (P<0.05). The ferritin level of the sJIA group was significantly higher than the KD, SIF, and JIA groups (P<0.05) but showed no significant difference compared to the ALL group (P>0.05). The sJIA group exhibited more instances of joint swelling and pain than other groups, and the difference was statistically significant (P<0.05) (Table 1).

**Table:**
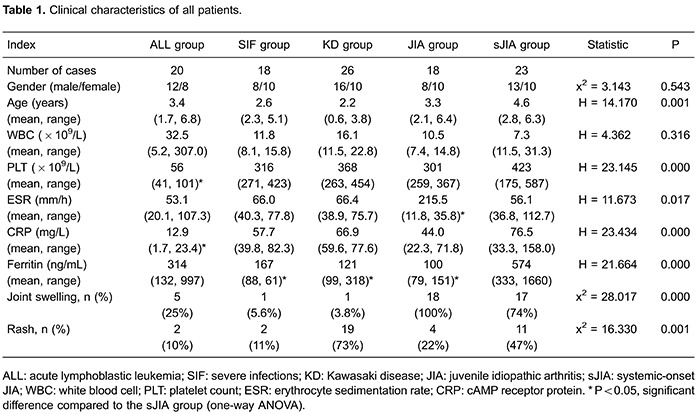

### Serum concentrations of IL-6, IL-18, S100A8, and S100A9

**Figure 1:**
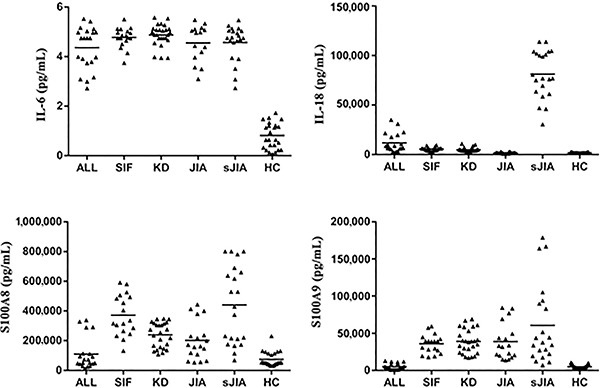
Concentrations of serum markers of all patients. Data are reported as means±SD. IL: interleukin; ALL: acute lymphoblastic leukemia; SIF: severe infections; KD: Kawasaki disease; JIA: juvenile idiopathic arthritis; sJIA: systemic-onset JIA; HC: healthy control.

The serum IL-6 levels in various disease groups were all significantly higher than the healthy control group (P<0.05), but the IL-6 concentration showed no significant difference between disease groups (P>0.05). However, the serum concentration of IL-18 showed significant differences between groups (F=24.18, P=0.000); the sJIA group showed a significantly higher IL-18 level than the normal control group (81,269±24,105 *vs* 1,930±302 pg/mL, P=0.000), ALL group (11,824±9,168 pg/mL, P=0.000), SIF group (5,643±1,935 pg/mL, P=0.000), KD group (4,985±2,170 pg/mL, P=0.000), and JIA group (1,818±644 pg/mL, P=0.000). The sJIA group showed a significantly higher S100A8 expression level than the control group (440,520±261,210 *vs* 74,551±48,372 pg/mL, P=0.000) but showed no significant difference compared to the SIF group (372,050±137,260 pg/mL, P=0.304), KD group (275,670±99,822 pg/mL, P=0.053), and ALL group (334,540±151,780 pg/mL, P=0.100). The serum S100A8 concentration was significantly increased in the sJIA group compared to the JIA group (201,980±129,020 pg/mL, P=0.001). S100A9 expression in the sJIA group was significantly higher than that in the control group (60,741±53,847 *vs* 5,081±2,809 pg/mL, P=0.000) and ALL group (5,204±4,205 pg/mL, P=0.0000) but showed no significant difference compared to the SIF group (35,942±12,517 pg/mL, P=0.052), KD group (31,904±16,135 pg/mL, P=0.051), and JIA group (38,681±24,496 pg/mL, P=0.059) (Figure 1).

### ROC curve analysis

A ROC comparison of IL-18, S100A8 and S100A9 was conducted to determine the predictive values of these markers in differentiating sJIA from other diseases. When comparing the ALL and sJIA groups, the area under the curve (AUC) of IL-18, S100A8, and S100A9 was greater than 90%, which suggested a remarkable sensitivity and specificity of these three biomarkers in differentiating sJIA from ALL. Among the markers, IL-18 showed a maximum AUC of 0.995, indicating a relatively better predictive value. When comparing sJIA to SIF, the AUC of IL-18 was 1. When the cut off value of IL-18 was set to 2.00×10^4^ pg/mL, IL-18 exhibited 100% sensitivity and specificity in distinguishing sJIA from SIF patients. When comparing the KD and sJIA groups, S100A8 and S100A9 showed no predictive value in differentiating sJIA from KD (P>0.05). When the cut off value of IL-18 was set to 2.08×10^4^ pg/mL, S100A8 and S100A9 exhibited 100% sensitivity and specificity in distinguishing sJIA from KD. However, S100A8 and S100A9 showed no predictive value in differentiating between sJIA and JIA (P>0.05). When the cut off value of IL-18 was ≥1.68×10^4^ pg/mL, its sensitivity and specificity in distinguishing sJIA from JIA was 100% (Table 2).

**Table:**
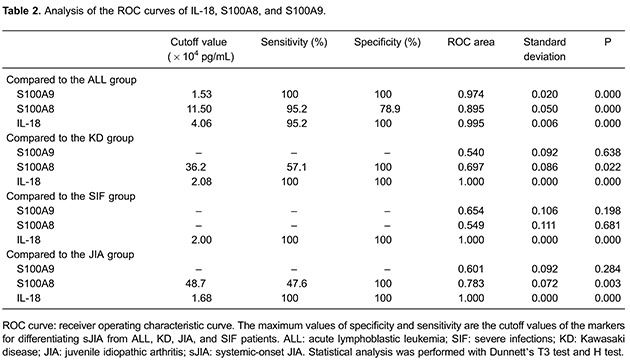

**Figure 2:**
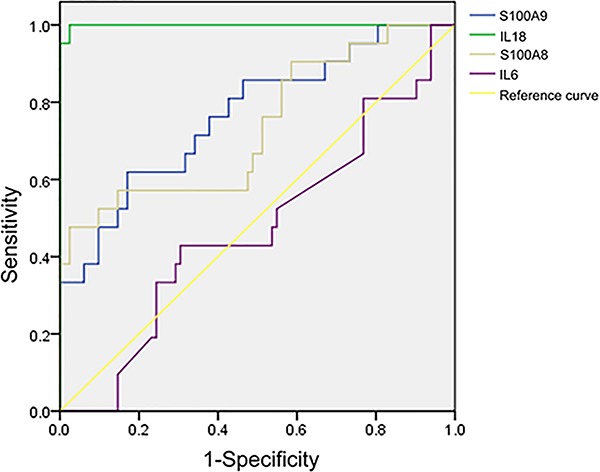
ROC curves of S100A9, IL-18, S100A8, and IL-6 for predicting systemic-onset juvenile idiopathic arthritis patients.

Further comparison of the predictive values between S100A9, IL-18, S100A8, and IL-6 for sJIA showed that IL-18 had a larger AUC than the other markers, indicating a relatively higher sensitivity and specificity. The predictive value of S100A8 and S100A9 for sJIA had a high sensitivity but low specificity. IL-6 showed no diagnostic value for prediction of sJIA (P>0.05; Figure 2, Table 3).

**Table:**
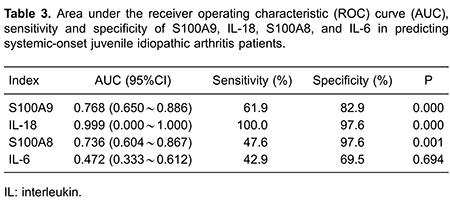

### Serum IL-6 and IL-18 concentrations in sJIA patients and correlations with joint damage

**Figure 3:**
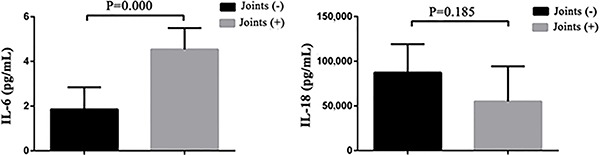
Relationship between serum concentrations of IL-6 and IL-18 and the presence (+) or absence (-) of joint damage. Data are reported as means±SD. IL: interleukin. One-way ANOVA was used for statistical analyses.

Among patients with sJIA, the IL-6 serum concentrations of those who had joint damage (n=17) were significantly higher than those without damage (n=6; 4.51±0.92 *vs* 1.83±0.97 pg/mL, t=-6.311, P=0.000). However, the serum concentrations of IL-18 showed no significant difference between the two groups (87,251±31,837 *vs* 55,122±39,473 pg/mL, t=1.420, P=0.185; Figure 3).

## Discussion

The clinical manifestations of sJIA are complicated. Symptoms such as fever, increased hemogram parameters, enlargement of the liver and spleen, rash, and other characteristics of sJIA can be found in other diseases. The misdiagnosis of sepsis, malignant tumor, or other febrile illnesses often occurs (6). These conditions delay the diagnosis and treatment of both sJIA and other diseases such as infection and tumors. Therefore, it is necessary to identify sensitive and specific biomarkers to assist in the diagnosis of sJIA. Because sJIA is an auto-inflammatory disease, we aimed to study whether the differential levels of inflammatory cytokines could help distinguish sJIA from other febrile diseases.

Pediatric patients with sJIA showed significantly increased acute stage inflammatory indexes, such as increased CRP and ferritin levels, compared to other disease groups (7). The present study also found that the CRP level was elevated in the other febrile disease groups, except for the ALL group. The ferritin level showed no difference between the sJIA and ALL groups, which indicated that routine inflammatory indexes have obvious limitations in the diagnosis of sJIA. Previous studies have indicated that IL-6 plays an important role in the pathogenesis of sJIA (8). IL-6 levels in the serum and synovial fluid are positively associated with the severity of joint damage. In addition, sJIA pediatric patients with increased IL-6 levels have responded well to treatment with an IL-6 receptor antagonist (9). The present study showed that the IL-6 level of the sJIA group was higher than that of the normal control group but exhibited no significant difference compared to the SIF and KD groups, which suggests that IL-6 cannot be used as a biomarker for the differential diagnosis of sJIA.

Previous studies have shown that the serum levels of S100A8 and S100A9 in patients with active sJIA are significantly increased. These studies attempted to use S100A8 and S100A9 as biological markers for the differential diagnosis of sJIA from other febrile diseases (10,11). The S100A8 and S100A9 proteins belong to the calcium-binding protein family. In an inflammatory reaction, inflammatory cells such as monocytes, macrophages, and neutrophils are abnormally activated, which causes increased secretion of these two proteins. In local or systemic inflammatory reactions, tissue or serum S100 protein concentrations have been suggested to reflect disease activity (12–15). The present study showed that the S100A8 and S100A9 serum concentrations during the active period of sJIA were significantly increased compared to those of ALL and JIA patients. When the S100A8 serum concentration was ≥11.50×10^4^ pg/mL, the sensitivity in distinguishing sJIA from ALL was 95.2%, and the specificity was 78.9%. When the S100A9 serum concentration was ≥1.53×10^4^ pg/mL, the sensitivity and specificity of distinguishing between sJIA and ALL were both 100%. But S100A8 and S100A9 showed a low sensitivity and specificity in differentiating sJIA from KD and JIA. When the cutoff point of S100A8 was ≥48.70×10^4^ pg/mL, the sensitivity of distinguishing sJIA from JIA was only 47.6%, and the specificity was 88.9%. When the cutoff point of S100A8 was ≥36.2×10^4^ pg/mL, the sensitivity in distinguishing KD from sJIA was 57.1%, and the specificity was 100%. Meanwhile, S100A8 and S100A9 could not distinguish between SIF and sJIA because the AUC of the ROC curve was less than 0.5, lacking differential diagnostic value. According to our data, S100A8 and S100A9 are not optimal as biological markers for the differential diagnosis of sJIA from other acute febrile diseases.

IL-18 is produced by activated macrophages and is an IFN-γ-inducible factor that stimulates inflammatory reactions and promotes the activation and proliferation of T cells and NK cells (16). Several studies have demonstrated that IL-18 levels are significantly elevated in the active period of sJIA and are associated with disease activity (17,18). However, IL-18 has not been reported as a serological marker for the diagnosis of sJIA. The results of this study showed that the expression of IL-18 in the sJIA group was significantly higher than that of the other disease groups. Remarkably, the sensitivity and specificity of IL-18 in differentiating the sJIA group from the JIA, SIF, and KD groups were all 100%. Recently, some scholars found that the serum concentrations of IL-18 in sJIA patients were significantly higher than those of atypical KD patients and proposed that the IL-18 serum concentration could be used as a basis for the differential diagnosis between the two diseases (19). In the present paper, the predictive value of markers for sJIA was further analyzed by ROC analysis. The results showed that the sensitivity of IL-18 in predicting sJIA was 100%, and the specificity was 97.6%, indicating the significance of the IL-18 serum concentration in the differential diagnosis of sJIA.

Our study was, to the best of our knowledge, the first to determine the elevated IL-18 levels as a potential diagnostic marker for sJIA. However, we also have to note certain limitations of our work. The present study involved a small sample of patients and limited disease types. Other diseases, such as systemic lupus erythematosus, inflammatory bowel disease, and autoimmune lymphoproliferative syndrome, also exhibit elevated serum IL-18 concentrations (20–22). Therefore, studies with a larger number of cases and disease types are needed. In spite of limitations, the present study revealed that the IL-18 level was significantly increased in sJIA patients, and the sensitivity and specificity of IL-18 in differentiating sJIA from SIF, JIA, and KD were both 100% based on ROC curve analysis. Thus, IL-18 may be used as a biomarker for the differential diagnosis of this disease.

## References

[1] Ravelli A, Martini A (2007). Juvenile idiopathic arthritis. Lancet.

[2] Petty RE, Southwood TR, Manners P, Baum J, Glass DN, Goldenberg J (2004). International League of Associations for Rheumatology classification of juvenile idiopathic arthritis: second revision, Edmonton, 2001. J Rheumatol.

[3] Foell D, Roth J (2004). Proinflammatory S100 proteins in arthritis and autoimmune disease. Arthritis Rheum.

[B04] Kessel C, Holzinger D, Foell D (2013). Phagocyte-derived S100 proteins in autoinflammation: putative role in pathogenesis and usefulness as biomarkers. Clin Immunol.

[5] Martini A (2012). Systemic juvenile idiopathic arthritis. Autoimmun Rev.

[6] Gurion R, Lehman TJ, Moorthy LN (2012). Systemic arthritis in children: a review of clinical presentation and treatment. Int J Inflam.

[7] Ling XB, Park JL, Carroll T, Nguyen KD, Lau K, Macaubas C (2010). Plasma profiles in active systemic juvenile idiopathic arthritis: Biomarkers and biological implications. Proteomics.

[8] Mellins ED, Macaubas C, Grom AA (2011). Pathogenesis of systemic juvenile idiopathic arthritis: some answers, more questions. Nat Rev Rheumatol.

[9] Herlin T (2009). Tocilizumab: The evidence for its place in the treatment of juvenile idiopathic arthritis. Core Evid.

[10] Holzinger D, Frosch M, Kastrup A, Prince FH, Otten MH, Van Suijlekom-Smit LW (2012). The Toll-like receptor 4 agonist MRP8/14 protein complex is a sensitive indicator for disease activity and predicts relapses in systemic-onset juvenile idiopathic arthritis. Ann Rheum Dis.

[11] Frosch M, Ahlmann M, Vogl T, Wittkowski H, Wulffraat N, Foell D (2009). The myeloid-related proteins 8 and 14 complex, a novel ligand of Toll-like receptor 4, and interleukin-1beta form a positive feedback mechanism in systemic-onset juvenile idiopathic arthritis. Arthritis Rheum.

[12] Perera C, McNeil HP, Geczy CL (20160). S100 Calgranulins in inflammatory arthritis. Immunol Cell Biol.

[B13] Vogl T, Tenbrock K, Ludwig S, Leukert N, Ehrhardt C, van Zoelen MA (2007). Mrp8 and Mrp14 are endogenous activators of Toll-like receptor 4, promoting lethal, endotoxin-induced shock. Nat Med.

[B14] Roth J, Vogl T, Sorg C, Sunderkotter C (2003). Phagocyte-specific S100 proteins: a novel group of proinflammatory molecules. Trends Immunol.

[15] Frosch M, Strey A, Vogl T, Wulffraat NM, Kuis W, Sunderkotter C (2000). Myeloid-related proteins 8 and 14 are specifically secreted during interaction of phagocytes and activated endothelium and are useful markers for monitoring disease activity in pauciarticular-onset juvenile rheumatoid arthritis. Arthritis Rheum.

[16] Novick D, Kim S, Kaplanski G, Dinarello CA (2013). Interleukin-18,more than a Th1 cytokine. Semin Immunol.

[17] Lotito AP, Campa A, Silva CA, Kiss MH, Mello SB (2007). Interleukin 18 as a marker of disease activity and severity in patients with juvenile idiopathic arthritis. J Rheumatol.

[18] de Jager W, Hoppenreijs EP, Wulffraat NM, Wedderburn LR, Kuis W, Prakken BJ (2007). Blood and synovial fluid cytokine signatures in patients with juvenile idiopathic arthritis: a cross-sectional study. Ann Rheum Dis.

[19] Takahara T, Shimizu M, Nakagishi Y, Kinjo N, Yachie A (2015). Serum IL-18 as a potential specific marker for differentiating systemic juvenile idiopathic arthritis from incomplete Kawasaki disease. Rheumatol Int.

[20] Xu WD, Pan HF, Ye DQ (2013). Association of interleukin-18 and systemic lupus erythematosus. Rheumatol Int.

[B21] Kanai T, Kamada N, Hisamatsu T (2013). Clinical strategies for the blockade of IL-18 in inflammatory bowel diseases. Curr Drug Targets.

[22] Teachey DT (2014). Targeting cytokines in ALPS: it's FAShionable. Blood.

